# Galectin-12 in Cellular Differentiation, Apoptosis and Polarization

**DOI:** 10.3390/ijms19010176

**Published:** 2018-01-07

**Authors:** Lei Wan, Ri-Yao Yang, Fu-Tong Liu

**Affiliations:** 1 School of Chinese Medicine, China Medical University, Taichung 40402, Taiwan; lei.joseph@gmail.com; 2 Department of Biotechnology, Asia University, Taichung 41354, Taiwan; 3 Department of Gynecology, China Medical University Hospital, Taichung 40402, Taiwan; 4 Research Center for Chinese Medicine & Acupuncture, China Medical University, Taichung 40402, Taiwan; 5 Department of Molecular and Cellular Oncology, University of Texas MD Anderson Cancer Center, Houston, TX 77030, USA; ryang@mdanderson.org; 6 Institute of Biomedical Sciences, Academia Sinica, Taipei 11529, Taiwan; 7 Department of Dermatology, University of California-Davis, School of Medicine, Sacramento, CA 95816, USA

**Keywords:** galectin-12, differentiation, adipogenesis, cellular plasticity

## Abstract

Galectin-12 is a member of a family of mammalian lectins characterized by their affinity for β-galactosides and consensus amino acid sequences. The protein structure consists of a single polypeptide chain containing two carbohydrate-recognition domains joined by a linker region. Galectin-12 is predominantly expressed in adipose tissue, but is also detected in macrophages and other leukocytes. Downregulation of galectin-12 in mouse 3T3-L1 cells impairs their differentiation into adipocytes. Conversely, overexpression of galectin-12 in vitro induces cell cycle arrest in G1 and apoptosis. Upregulation of galectin-12 and initiation of G1 cell cycle arrest are associated with driving pre-adipocytes toward terminal differentiation. Galectin-12 deficiency increases insulin sensitivity and glucose tolerance in obese animals. Galectin-12 inhibits macrophage polarization to the M2 population, enhancing inflammation and decreasing insulin sensitivity in adipocytes. Galectin-12 also affects myeloid differentiation, which is associated with chemotherapy resistance. In addition to highlighting the above-mentioned aspects, this review also discusses the potential clinical applications of modulating the function of galectin-12.

## 1. The Family of Galectins

Galectins are known for their β-galactoside-binding activities and, to date, 17 galectins have been identified. Galectins share consensus sequences in the carbohydrate-recognition domains (CRDs). Galectin-1, -2, -5, -7, -10, -11, -13, -14, -15, -16, and -17 are known as one-CRD-type galectins. Galectin-4, -6, -8, -9, and -12 are two-CRD-type galectins, containing two homologous CRDs in a single polypeptide chain. Galectin-3, the only chimeric galectin, contains one CRD and a non-lectin region comprising proline- and glycine-rich short tandem repeats. Some galectins are widely distributed among different tissues, whereas others are tissue-specific. One-CRD galectins often exist as homodimers, whereas two-CRD galectins have two carbohydrate-binding sites. In addition, galectin-3 can form oligomers, which exhibit multivalent carbohydrate-binding activity. Moreover, the carbohydrate-binding specificities and affinities differ between galectin family members [1,2].

Galectins do not contain a classical leader sequence and mainly exist as intracellular proteins. However, they can also be detected on the cell surface and in the extracellular space [1,2], although how these proteins are transported to these areas remains unknown. Galectins do not have specific cell-surface receptors, but bind to various glycoproteins via their carbohydrate moieties. Mostly through the use of recombinant proteins and cultured single cell types, galectins have been shown to exhibit various in vitro activities by interacting with cell-surface glycoproteins or extracellular matrix proteins in a carbohydrate-dependent manner. However, these may not represent the natural biological functions of endogenous galectins. Whether these activities can occur in multi-cellular situations in vivo is uncertain. Moreover, the concentrations of recombinant proteins used to demonstrate extracellular functions might not be reachable under physiological or pathological conditions. Additionally, many studies have revealed intracellular biological activities of galectins, some of which are independent of carbohydrate binding. Indeed, galectins bind to a number of intracellular signaling molecules to regulate signal transduction [1,2]. Galectins have been shown to participate in cell adhesion, migration, and growth, and have been implicated in the pathogenesis of a variety of diseases, including cancer initiation, progression, and metastasis [3,4]. Importantly, galectins also influence the activation and regulation of both innate and adaptive immune responses.

## 2. Biology of Galectin-12

**Figure 1:**
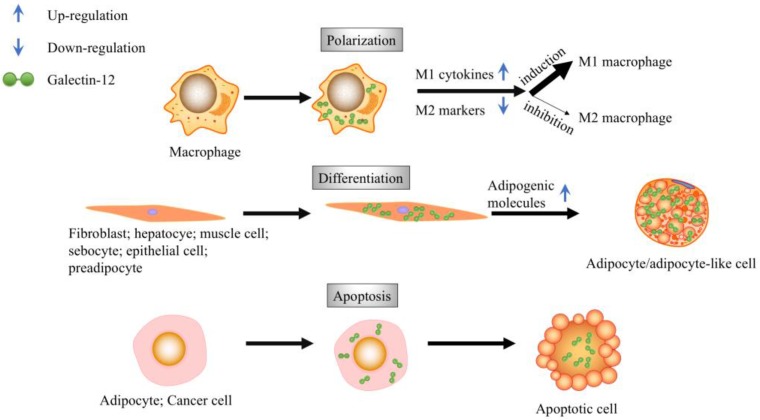
Biological functions of galectin-12. Galectin-12 is expressed in macrophages and promotes the M1 polarization of macrophages. Galectin-12 expression promotes apoptosis in adipocytes, as well as cervical cancer cells. Galectin-12 expression promotes cell differentiation into adipocytes or adipocyte-like cells.

Galectin-12 was first discovered in 2001 by two independent groups [5,6]. This protein contains two CRDs joined by a linker region in a single polypeptide chain. Northern blot analysis revealed that galectin-12 is predominantly expressed in adipose tissue. It is also detected at low levels in the heart, pancreas, spleen, thymus, and peripheral blood leukocytes [6]. The amino acid sequence of galectin-12 shows considerable variation when compared with the consensus sequences of galectin family proteins. Comparison of the N-terminal CRD protein sequence with other CRDs from galectins 1–11 revealed 32–43% (an average of 38.8%) identity; however, the sequence identity of the C-terminal CRD with these other CRDs is 28–41% (an average of 33.7%). In addition, the C-terminal CRD of galectin-12 exhibits significant differences from other members of the galectin family in the conserved amino acid residues important for binding to β-galactosides. The amino acids matching Asn-160, Arg-162, Asn-174, and Glu-184, which are important residues for carbohydrate binding in galectin-3, are changed to Pro-251, Thr-253, Arg-261, and Lys-272 in the C-terminal CRD of galectin-12. The binding of galectin-12 to lactosyl-agarose is much weaker than that of galectin-8 [5]. Although the possibility of galectin-12 secretion and binding to certain physiologically or pathologically relevant glycan targets under some specific conditions cannot be totally ruled out, the data above are consistent with its being a galectin that functions intracellularly, independent of glycan binding. The biological functions of galectin-12 have only started to emerge; in this review, the bioactivities of galectin-12 in apoptosis and differentiation are discussed (Figure 1).

## 3. Galectin-12 Regulates Cell Growth and Apoptosis

The expression of galectin-12 is tightly regulated, as evidenced by the fact that (1) the start codon of galectin-12 is known to be a weak initiation site for translation according to the Kozak rule, and (2) the 3′ untranslated region of galectin-12 mRNA consists of an AU (AUUUA)-rich motif, suggesting mRNA instability. The AU-rich motif has been detected in many proto-oncogenes, transcription factors, and cytokines, which play critical roles in cell growth and differentiation [6]. Consistently, galectin-12 has been shown to play a role in cell cycle regulation. Jurkat T cells synchronized at the G1/S boundary or G1 phase showed increased levels of galectin-12; moreover, overexpression of galectin-12 in the human cervical cell line HeLa caused cell cycle arrest at G1 phase [6]. Furthermore, ectopic expression of galectin-12 induced apoptosis in COS-1 cells. The number of apoptotic cells was also elevated in the adipose tissue of Zucker rats treated with troglitazone, which induces the expression of galectin-12. A positive correlation between galectin-12 mRNA level and the number of apoptotic cells in adipose tissue has been observed [5]. Overexpression of galectin-12 in vitro induces cell cycle arrest in G1 and apoptosis. Moreover, upregulation of galectin-12 and initiation of G1 cell cycle arrest are associated with driving pre-adipocytes towards terminal differentiation. In acute myeloid leukemia, the expression level of galectin-12 is downregulated (88.7% downregulation compared to normal controls) and patients with lower *galectin-12* levels have a lower survival rate compared to those with higher galectin-12 levels [7]. These results suggest anti-proliferative and pro-apoptotic function of galectin-12, although further mechanistic studies are required to establish which cell death and proliferation pathways are involved, and under what physiological/pathological circumstances such function of galectin-12 is evoked.

## 4. Galectin-12 in Cellular Differentiation

**Figure 2:**
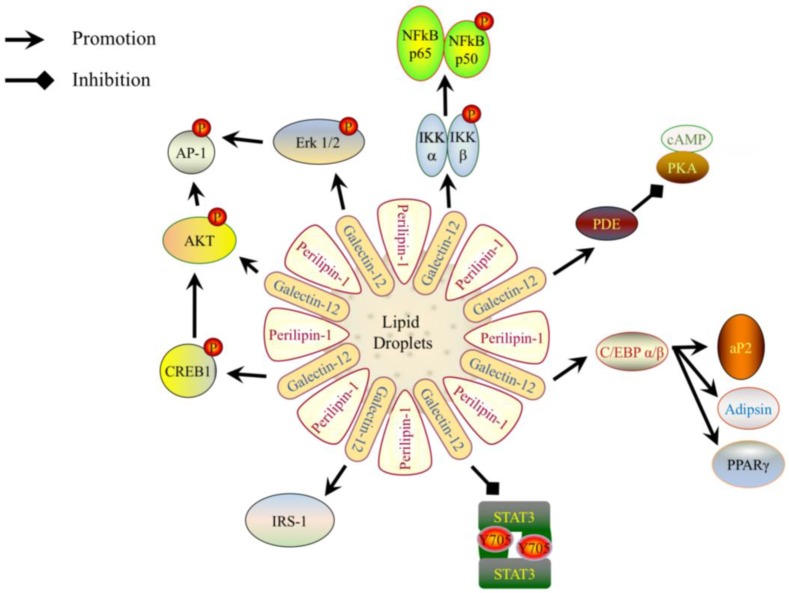
A schematic representation of how galectin-12 modulates the signaling pathways to elicit its biological activities. Galectin-12 is localized on lipid droplets and is co-localized with Perilipin-1. Galectin-12 promotes the expression of the adipogenic transcription factors Ccaat-enhancer-binding protein α (C/EBPα), C/EBPβ, and peroxisome proliferator-activated receptor γ (PPARγ), as well as the early adipocyte differentiation marker adipocyte protein 2 (aP2), and late adipocyte differentiation marker adipsin. Galectin-12 also enhances the activation of protein kinase B (Akt), extracellular receptor signal-related kinase (ERK) and cyclic AMP-responsive element-binding protein 1 (CREB1), which in turn promote activator protein 1 (AP-1). The decreased size of adipocytes in galectin-12-deficient mice is due to increased lipolysis. This is because galectin-12 negatively regulates phosphodiesterase (PDE) activity and cyclic AMP (cAMP) levels, which is upstream of protein kinase A (PKA) activity. Galectin-12 also promotes expression of insulin receptor substrate 1 (IRS-1), as well as activation of IκB kinases (IKK)α/β; the latter in turn enhances the activation of nuclear factor-κB (NF-κB). Finally, galectin-12 suppresses the activation of signal transducer and activator of transcription 3 (STAT3).

The level of galectin-12 in 3T3-L1 cells is low in the subconfluent state but increased in a state of growth arrest when cells reach confluence. Galectin-12 level was further increased when confluent 3T3-L1 cells were treated with adipogenic hormones to promote differentiation into adipocytes. Galectin-12 is required for adipocyte differentiation, as downregulation of galectin-12 by siRNA inhibited the adipocyte differentiation of 3T3-L1 cells initiated by adipogenic hormones [8]. Expression levels of the adipogenic transcription factors Ccaat-enhancer-binding protein α (C/EBPα), C/EBPβ, and peroxisome proliferator-activated receptor γ (PPARγ), as well as the early adipocyte differentiation marker adipocyte protein 2 (aP2), and late adipocyte differentiation marker adipsin, were downregulated in 3T3-L1 cells treated with galectin-12 siRNA (Figure 2). The activation of protein kinase B (Akt), extracellular receptor signal-related kinase (ERK), and cyclic AMP-responsive element-binding protein 1 (CREB1), which are important signaling molecules in the adipogenic signaling pathway, was also inhibited (Figure 2) [8]. 

Galectin-12 has been shown to bind to VPS13C, a member of the VPS13 family of proteins, which are associated with vacuolar protein sorting (VPS), and are highly conserved throughout eukaryotic evolution. The protein has been shown to be required for galectin-12 stability. In VPS13C knockdown cells, galectin-12 is degraded through the lysosomal pathway and adipocyte differentiation is suppressed [9]. Whether this protein otherwise mediates the function of galectin-12 is unknown. It is also not known whether galectin-12 interacts with VPS13C in other cell types. Loss of function of VPS13C is known to cause an early-onset form of Parkinson disease with a distinct phenotype of severe and rapid progression and a cellular manifestation of mitochondrial dysfunction and increased mitophagy [10]. Expression of galectin-12 in normal or diseased brain has not been carefully characterized, and whether it is involved in this disease remains to be clarified. 

Galectin-12 is predominantly located on large lipid droplets. Galectin-12-deficient mice exhibited a ~40% reduction in whole body lipid content with smaller adipocyte sizes compared with wild-type mice. Low adiposity in galectin-12-deficient mice are due to increased lipolysis, which is initiated by defective phosphodiesterase activity to increase cyclic AMP (cAMP) levels. Increased cAMP subsequently increases protein kinase A (PKA) activity and the activity of hormone-sensitive lipase, which functions with adipocyte triglyceride lipase to promote the hydrolysis of lipids stored in adipocytes. The metabolic rate of galectin-12-deficient mice is high, as indicated by a higher oxygen consumption in galectin-12-deficient adipocytes than in wild-type cells [11,12]. 

In addition, galectin-12 has also been suggested as an important molecule regulating glucose homeostasis, as galectin-12 downregulation lowered the expression of insulin receptor, as well as insulin receptor substrate 1 (IRS-1) (Figure 2). Galectin-12 deficiency increases insulin sensitivity and glucose tolerance in obese animals [11]. Collectively, these data show that galectin-12 is important in adipose tissue fatty acid metabolism and whole-body energy metabolism, and may be a promising therapeutic target in combating metabolic disorders [13]. 

Galectin-12 was also detected in sebaceous glands of human scalp skin and subsequently confirmed to be expressed in sebocytes, which are sebum-secreting cells. C/EBPα and PPARγ, two molecules regulated by galectin-12, were shown to have important roles in sebocyte differentiation. This suggests that galectin-12 is important in sebocyte differentiation and in the biological function of sebocytes [14]. The differentiation of acute promyelocytic leukemia cells was investigated by Xue et al. [15], who found that galectin-12 is involved in all-trans retinoic acid-induced granulocytic differentiation. Downregulation of galectin-12 promoted all-trans retinoic acid-induced neutrophil differentiation, but impeded the formation of lipid droplets in terminally differentiated neutrophils, suggesting that lipogenesis and other aspects of myeloid differentiation can be independently regulated by galectin-12. Consistently, downregulation of galectin-12 interferes with the lipogenic pathway by lowering the levels of lipogenic transcription factors C/EBPα, C/EBPβ, PPARγ, as well as the lipogenic signaling pathway by inhibiting the activation of CREB1 and ERK (Figure 2).

## 5. Galectin-12 as a Modulator of Cell Plasticity?

Differentiation is not a permanent event, but rather is associated with a great deal of plasticity [16]. In our previous study, we found that galectin-12 was expressed in macrophages. Ablation of galectin-12 did not affect the differentiation of bone marrow cells into macrophages, but decreased phagocytic activity against *Escherichia coli* and reduced the secretion of nitric oxide. Ablation of galectin-12 also resulted in polarization of macrophages to the M2 subset, as indicated by increased expression levels of M2 markers, namely resistin-like molecule beta and chitinase 3-like protein 3, as well as reduced expression levels of numerous M1 pro-inflammatory cytokines. The lowered expression of pro-inflammatory cytokines in macrophages by galectin-12 deletion was due to lowered activation of IKKα/β, Akt, and extracellular receptor signal-related kinase, which in turn reduced the activation of nuclear factor-κB and activator protein 1. STAT3 activation was significantly higher in Gal12^−/−^ macrophages induced by lipopolysaccharide, which corresponded to higher levels of interleukin-10 (Figure 2). Adipocytes showed higher insulin sensitivity when treated with Gal12^−/−^ macrophage-conditioned media than those treated with Gal12^+/+^ macrophage-conditioned media. Galectin-12 negatively regulates macrophage polarization into the M2 population, causing enhanced inflammation and decreased insulin sensitivity in adipocytes [17]. The reduced inflammation in Gal12^−/−^ macrophages may also result from an increase in cAMP level which is mediated by PKA-A kinase-anchoring protein (AKAP) 95-p105 pathway [18]. Galectin-12 has been shown to affect inflammation-related signaling pathways, which suggests that galectin-12 may serve as an adaptor protein that brings signaling molecules in close proximity to promote signal transduction.

Plasticity allows cells to switch morphology and function in response to internal or external stimulus, which promotes differentiation from one phenotype to another [16]. The functions of galectin-12 suggest that this molecule serves as a cell plasticity modulator to promote cellular reprogramming to change the biological function. Lipid droplets are universal organelles composed of neutral lipids as an energy source or for membrane synthesis [19]. Fatty liver is characterized by the accumulation of fat in hepatocytes. Large lipid droplets may eventually displace the nucleus of hepatocytes, which is a characteristic of adipocytes with large lipid droplets. Excessive lipids in hepatocytes may confer hepatocytes with adipocyte-like activities. The lipogenesis genes coding for C/EBPα, C/EBPβ, and PPARγ are elevated in the liver with excess lipid accumulation [20,21]. The levels of inflammatory cytokines tumor necrosis factor α [22], interleukin-6 [23], and monocyte chemoattractant protein-1 [24] are also increased in fatty liver. The expression of galectin-12 promotes the secretion of inflammatory cytokines [11]. In our previous study, we found a very low level of galectin-12 in normal hepatocytes, but an increase in galectin-12 in the liver of C57BL/6J mice with steatosis induced by high-fat diet. We hypothesized that galectin-12 is not expressed in hepatocytes under normal conditions (with small lipid droplet). However, under conditions of energy excess, lipid biosynthesis increases and lipid droplets are enlarged because of excessive fat, which promotes hepatocytes to differentiate into an adipocyte-like phenotype and induces the expression of galectin-12. It remains unknown whether the expression of galectin-12 is induced by excessive fat in hepatocytes or increased fatty acids in the blood stream, which confers hepatocytes with an adipocyte-like phenotype to store more energy. This condition may apply to several different cell types in obesity, in which many cells store large lipid droplets.

## 6. Conclusions

Galectin-12 has been shown to play important roles in regulating growth, differentiation, and lipolysis, as well as in modulating inflammation. However, many questions remain unanswered. For example, whether galectin-12 is involved in cellular plasticity is unclear. In many cases, endogenous galectin-12 is upregulated in a way that correlates with lipid accumulation, suggesting that a hydrophobic environment may be required for its proper localization and function. VPS13C has been identified as a galectin-12-binding protein required for galectin-12 protein stability by preventing its degradation through the lysosomal pathway [9]. While the function of galectin-12 in adipocytes is reasonably well studied, much remains to be explored about its roles in other cell types. Further studies on the regulation of galectin-12 expression and downstream signaling pathways will yield a more complete picture on the functions of this important protein.
